# Motor Unit Number Index (MUNIX) in Control Children: Reference Values and Reliability

**DOI:** 10.1002/mus.28470

**Published:** 2025-07-21

**Authors:** Christophe Boulay, Emilien Delmont, Frédérique Audic, Cécile Halbert, Sébastien Pesenti, Brigitte Chabrol, Béatrice Desnous, Shahram Attarian

**Affiliations:** ^1^ Reference Center for Neuromuscular Diseases and ALS Timone University Hospital Aix‐Marseille University Marseille France; ^2^ Gait Laboratory, Pediatric Orthopaedic Surgery Department Timone Children Hospital Marseille France; ^3^ CNRS, ISM UMR 7287 Aix‐Marseille University Marseille France; ^4^ UMR 7286, Medicine Faculty Aix‐Marseille University Marseille France; ^5^ Neuropediatric Department, Children Timone University Hospital Aix‐Marseille University Marseille France; ^6^ Inserm UMR S 910, Medical Genetics and Functional Genomics Aix‐Marseille University Marseille France

**Keywords:** biomarker, CMAP, control children, MUNIX, MUSIX

## Abstract

**Introduction/Aims:**

The motor unit number index (MUNIX) is recognized as a reliable electrophysiological biomarker, and reference values are available for healthy adults but not for children. The aim of this study was to determine reference MUNIX values in healthy children.

**Methods:**

Sixty control children (28 females) were grouped as under 5 years (n_1_ = 16), 5–10 years (n_2_ = 15), 10–15 years (n_3_ = 20), and 15–17 years (n_4_ = 9). MUNIX measurements were performed by an experienced and an inexperienced investigator in the abductor digiti minimi (ADM) and abductor pollicis brevis (APB) on both sides when possible.

**Results:**

The APB MUNIX increased by 6.5 [95% confidence interval, 3.2–9.7] per year of age, 1.0 [0.5–1.5] per cm, and 1.7 [0.4–4.0] per kg (*p* < 0.001 in each case), but was not independently associated with either of these three variables in multivariable analysis. The APB compound muscle action potential (CMAP) increased by 0.42 [0.30–0.52] per year of age, 0.06 [0.04–0.08] per cm, and 0.11 [0.07–0.14] per kg (*p* < 0.001 in each case), and increased with age independently of height and weight at borderline significance (*p* = 0.065). Measurement reproducibility was high for the experienced investigator, but lower between the experienced and the inexperienced investigator.

**Discussion:**

The high intra‐investigator reproducibility for the experienced investigator indicates that these parameters are useful electrophysiological biomarkers for disease progression and potential treatment response.

**Abbreviations**
ADM: abductor digiti minimi
APB: abductor pollicis brevis
BMI: body mass index
CMAP: compound muscle action potential
ICC: intraclass correlation coefficient
ICMUC: ideal case motor unit count
MUNIX: motor unit number index
MUSIX: motor unit size index
SIP: surface interference pattern

## 1 Introduction

Motor unit number index (MUNIX) is an electrophysiological technique based on measurements of the compound muscle action potential (CMAP) and surface electromyography interference patterns [1, 2] and is generally simpler and faster to perform than traditional motor unit number estimation techniques [3, 4, 5, 6, 7, 8, 9, 10]. The main parameter obtained is the MUNIX itself, but this can be combined with the CMAP to obtain the motor unit size index (MUSIX). In patients with motor neuron diseases, MUNIX is a reliable biomarker of motor neuron loss while MUSIX is a measure of compensatory reinnervation. MUNIX is an established technique in adults and reference values are available for healthy subjects. No such data are available in children; however, because of misgivings regarding the feasibility of the technique in young children [11] and the potential variability of the measures with age. However, two recent studies have demonstrated the feasibility and value of MUNIX as a potential biomarker of disease progression in children with spinal muscular atrophy [12, 13], emphasizing the need for larger pediatric studies to obtain age‐specific reference values and evaluate the reproducibility of the technique in regular clinical practice.

The aims of this study were therefore to measure MUNIX and MUSIX in a large group of control children of different ages and evaluate the intra‐ and inter‐observer reproducibility of these parameters.

## 2 Methods

### 2.1 Study Design

This prospective single‐center study was conducted at the Timone hospital in Marseille, France (a national reference center for neuromuscular diseases). The study was approved by the institutional review board (registration number, PADS21‐103). Informed consent was obtained from the parents of all participants. The participants were 60 children referred to our center on suspicion of neuromuscular disease but in whom no neuromuscular disorder was found. Children with neurodevelopmental disorders were excluded. All participants underwent clinical examination and electrodiagnostic testing between June 2018 and December 2021.

### 2.2 Data Collection

The participants' sex, age, height, weight, and body mass index (BMI) were collected. Heights and weights were converted to height‐for‐age and weight‐for‐age *Z*‐scores according to WHO standards. Muscle strength was evaluated via manual testing using the Medical Research Council (MRC) scale. The strength of the abductor digiti minimi (ADM) and abductor pollicis brevis (APB) muscles was measured on the right and left sides.

MUNIX measurements were performed on the ADM and APB muscles as described previously [1, 2, 4, 9, 14, 15]. The recording surface was 10 × 6 mm with an inter‐electrode distance of 10 mm. A smaller recording surface of 5 × 3 mm was used in cases (smallest children) where the electrodes could not be attached 10 mm apart. The corresponding nerves underwent supramaximal distal stimulation to achieve maximal CMAP amplitude with minimum rise time and a sharp negative takeoff. The recordings were assessed in a 300‐ms window with a filter setting of 3–3000 Hz. Ten surface interference patterns (SIPs) with different levels of contraction intensity (ranging from minimal to maximal) were recorded. The size of the force increment was estimated from experience and modulated by resistance provided by the examiner and monitored using the amplitude and form of the SIP [16, 17]. The data were exported from a Dantec Keypoint G4 electromyography workstation (Natus Medical Inc., Pleasanton, CA, USA) to an Excel file designed to calculate the MUNIX and MUSIX from

$\text{MUNIX}=A\times {\left(20\right)}^{\alpha }$with A and α determined by linear regression of logarithmic transformation of the relationship between the ideal case motor unit count (ICMUC) and the SIP area

$\text{ICMUC}=A\times {\left(\mathrm{SIP}\;\text{area}\right)}^{\alpha }$

The MUSIX was calculated by dividing the MUNIX by the CMAP amplitude [2]. SIP epochs were accepted if the SIP area was greater than 20 mV/ms, the ICMUC was below 100, and the SIP area/CMAP area was greater than 1 [2].

MUNIX, CMAP amplitude and MUSIX values were obtained for the ADM and APB muscles on both sides when possible. A game‐like approach was used to obtain the measurements. If children were uncooperative, measurements were only performed on their stronger side. Younger children were allowed to sit comfortably in the arms of their parents while the measurements were taken. However, because of time constraints, all measurements could not be obtained for all participants.

MUNIX measurements were performed by an experienced investigator and an inexperienced investigator. The data were all analyzed by the experienced investigator (CB). Intra‐operator variability was evaluated in data measured by the experienced investigator for children two or three times per muscle. Inter‐operator variability was evaluated for the APB muscle in 12 children (once in two children, twice in eight children and three times in two children) and for the ADM muscle in eight children (once in one child and twice in seven children). The age distributions were, for the APB muscle, two 5–10‐year‐olds, six 11–15‐year‐olds, and four 16–17‐year‐olds; and for the ADM muscle, two 5–10‐year‐olds, four 11–15‐year‐olds, and two 16–17‐year‐olds. The electrodes and marks were removed from participants after each measurement, and any trace of electrode placement was cleaned.

### 2.3 Statistical Analysis

Continuous variables were expressed as median (interquartile range, IQR) and 5–95th percentiles. Categorical variables were expressed as number (percentage). Associations between MUNIX, MUSIX, and CMAP amplitude values as dependent variables, and age, weight, height, and sex were tested in univariable and multivariable regression analysis. Regression results were expressed as the regression coefficient and corresponding 95% confidence interval, along with the adjusted *R* ^2^ as a goodness‐of‐fit measure. Intra‐ and interrater variability for MUNIX, MUSIX, and CMAP amplitude values was quantified using the two‐way random, single‐measure intraclass correlation coefficient (ICC). ICC values range from 0 to 1, with one indicating perfect reproducibility, and values > 0.75 conventionally interpreted as indicating good reproducibility [18]. All statistical analyses were performed using SPSS ver. 20 (IBM Corp., Armonk, NY, USA). All tests were two‐sided, and results were considered statistically significant at *p* < 0.05.

## 3 Results

Patient characteristics are summarized in Table 1. The 60 participants consisted of 28 girls (46%) and 32 boys (54%). The children were compliant, and the level of tolerance was excellent. MUNIX measurements took 20–30 min to complete.

**TABLE 1:** Patient characteristics by age group.

	< 5 years	5–9 years	10–15 years	16–17 years	*p*
	*n* = 16	*n* = 15	*n* = 20	*n* = 9	
Female sex	8 (50%)	5 (33%)	7 (33%)	8 (89%)	0.028
WFA *z*‐score	0.0 (−1.4–1.0)	0.5 (−0.1–1.8)	−0.1 (−0.5–0.2)	0.6 (0.1–1.3)	0.084
HFA *z*‐score	0.5 (−0.4–0.9)	1.4 (0.1–2.1)	0.6 (−0.2–1.0)	−0.3 (−0.8–0.2)	0.14

*Note*: Results are reported as number (percentage) or mean (standard deviation).

Abbreviations: HFA, height‐for‐age; WFA, weight‐for‐age.

The sex ratio varied significantly between age groups (*p* = 0.028). Muscle strength results were normal for age in all groups. The median weight‐for‐age and height‐for‐age *Z*‐scores were close to zero in all groups (i.e., close to the population average), except for children aged 5–9 years, who were substantially taller than average for age.

MUNIX parameter values are listed by age group in Table 2 and regression results are presented in Table S1.

**TABLE 2:** Reference values by age group in control children.

	< 5 years		5–9 years		10–15‐years		16–17‐years	
	Median (IQR)	P5–P95	Median (IQR)	P5–P95	Median (IQR)	P5–P95	Median (IQR)	P5–P95
MUNIX								
APB (*n* = 16, 15, 20, 9)	124 (92–153)	59–196	170 (127–201)	107–224	182 (146–270)	80–326	217 (192–243)	128–272
ADM (*n* = 5, 12, 16, 8)	118 (117–139)	99–217	181 (147–229)	91–280	155 (135–193)	93–250	148 (137–164)	108–181
MUSIX								
APB (*n* = 16, 15, 20, 9)	45 (40–48)	29–71	52 (39–59)	31–70	52 (40–68)	32–86	52 (49–61)	42–74
ADM (*n* = 5, 12, 16, 8)	45 (41–55)	34–56	59 (44–62)	32–69	63 (53–75)	35–85	63 (53–68)	43–89
CMAP amplitude (mV)								
APB (*n* = 16, 15, 20, 9)	5.7 (4.1–7.5)	2.8–7.9	8.1 (6.7–9.0)	4.6–11.1	10.1 (7.0–11.4)	6.2–14.4	11.6 (10.2–12.6)	8.2–13.8
ADM (*n* = 5, 12, 16, 8)	6.6 (5.3–7.5)	4.3–7.6	9.0 (7.9–10.7)	6.1–12.6	9.4 (8.0–11.0)	6.5–12.3	9.3 (7.6–9.7)	6.9–11.1
MUNIX–CMAP amplitude	Pearson *ρ*		Pearson *ρ*		Pearson *ρ*		Pearson *ρ*	
APB (*n* = 16, 15, 20, 9)	0.67		0.44		0.59		0.67	
ADM (*n* = 5, 12, 16, 8)	0.72		0.54		0.45		0.09	

*Note*: Sample sizes for the age subgroups are reported in the corresponding order in parentheses for each row.

Abbreviations: ADM, abductor digiti minimi; APB, abductor pollicis brevis; CMAP, compound muscle action potential; MUNIX, motor unit number index; MUSIX, motor unit size index (μV); NA, not applicable.

**FIGURE 1:**
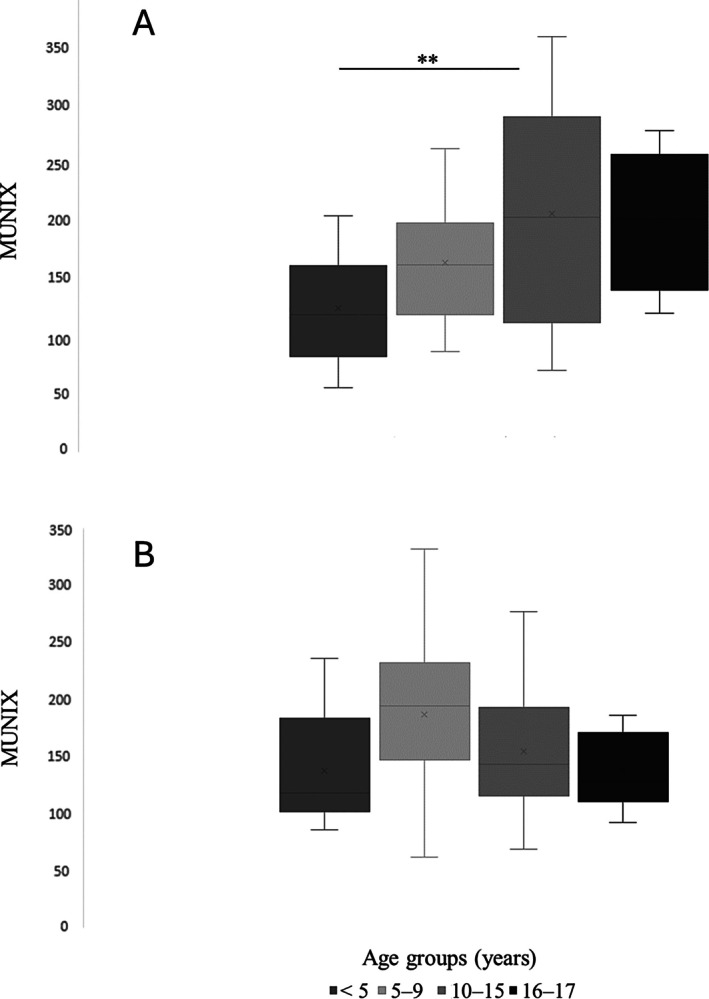
Box plot comparison of age groups in terms of (A) abductor pollicis brevis and (B) abductor digiti minimi motor unit index (MUNIX). **p < 0.05.

For the APB muscle, MUNIX values increased significantly with age, height, and weight (*p* < 0.001 in each case), but were not independently associated with either of these three variables in multivariable analysis (Table S1). The increasing trend with age was also observed between age groups (Figure 1A). MUSIX data were not well explained by a regression model (Table S1) and median values varied little between age groups (Table 2). CMAP amplitudes increased significantly with age, height, and weight in univariable analysis (*p* < 0.001 in each case), and in multivariable analysis, increased with age independently of height and weight at borderline significance (*p* = 0.065, Table S1). The 5–95 percentile ranges overlapped between age groups for all three variables (Table 2).

For the ADM muscle, MUNIX, MUSIX, and CMAP amplitude data were poorly explained by linear regression models (Table S1) and varied little between age groups (Table 2, Figure 1B). As described above for the APB muscle, the 5–95 percentile ranges overlapped between age groups for all three variables.

Intra‐rater reliability results are summarized in Table 3. APB measurements were obtained for 60 children (92 tests) and ADM measurements were obtained for 41 children (71 tests). Repeat measurements could not be performed on all children, so for the reliability analysis, the age groups considered were under‐fives and 5–9‐year‐olds together, and 10–17‐year‐olds. All intra‐rater ICC values were 0.75 or greater, indicating good reproducibility for all variables and age groups.

**TABLE 3:** Test–retest reliability of motor unit number estimation as assessed by intra‐rater intraclass coefficient (ICC) by age group in control children.

		< 5 years	5–9 years	10–17‐years	All
MUNIX	APB (*n* = 16, 31, 29)	**0.81**	**0.85**	**0.96**	**0.93**
	ADM (*n* = 5, 17, 24)	**0.75**	**0.96**	**0.92**	**0.92**
	APB + ADM^a^ (*n* = 5, 17, 24)	**0.86**	**0.93**	**0.95**	**0.95**
CMAP amplitude (mV)	APB (*n* = 16, 31, 29)	**0.97**	**0.97**	**0.91**	**0.96**
	ADM (*n* = 5, 17, 24)	**0.76**	**0.87**	**0.93**	**0.90**
	APB + ADM^a^ (*n* = 5, 17, 24)	**0.77**	**0.84**	**0.92**	**0.92**
MUSIX	APB (*n* = 16, 31, 29)	**0.85**	**0.80**	**0.80**	**0.79**
	ADM (*n* = 5, 17, 24)	**0.96**	**0.84**	**0.77**	**0.75**
	APB + ADM^a^ (*n* = 5, 17, 24)	**0.75**	**0.84**	**0.85**	**0.85**

*Note*: Sample sizes for the age subgroups are reported in the corresponding order in parentheses for each row. ICC values > 0.75, interpreted as indicating good reproducibility, are shown in bold.

Abbreviations: ADM, abductor digiti minimi; APB, abductor pollicis brevis; CMAP, compound muscle action potential; MUNIX, motor unit number index; MUSIX, motor unit size index (μV).

^a^ Sum scores were obtained by adding the values obtained for the two muscles.

Inter‐rater reliability results evaluating the reproducibility of the measurements between an experienced and inexperienced investigator are summarized in Table 4. APB measurements were compared for 24 children (24 tests) and ADM measurements for 15 children (15 tests). Measurements in under‐fives proved not to be feasible for the inexperienced investigator, so the age groups considered for this analysis were 5–9‐year‐olds and 10–17‐year‐olds. The inter‐rater ICCs for the younger age group were all below 0.75 and in most cases below 0.5, indicating poor reproducibility. The ICCs for the sum scores over both muscles were nevertheless higher (0.55 and 0.66, for MUNIX and CMAP amplitude, respectively). In 10–17‐year‐olds, 4/6 of the MUNIX and CMAP ICCs were greater than 0.75. The MUSIX ICCs were all well below 0.75.

**TABLE 4:** Test–retest reliability of motor unit number estimation as assessed by inter‐rater intraclass coefficient (ICC) by age group in control children.

		5–9 years	10–17‐years	All
MUNIX	APB (*n* = 2, 12)	0.42	**0.83**	**0.81**
	ADM (*n* = 2, 10)	0.10	0.65	0.55
	APB + ADM^a^ (*n* = 2, 10)	0.55	**0.78**	**0.76**
CMAP amplitude (mV)	APB (*n* = 2, 12)	0.12	**0.79**	**0.80**
	ADM (*n* = 2, 10)	0.19	**0.93**	**0.89**
	APB + ADM^a^ (*n* = 2, 10)	0.66	0.70	0.69
MUSIX	APB (*n* = 2, 12)	0.19	0.58	0.48
	ADM (*n* = 2, 10)	ND^b^	0.53	0.48
	APB + ADM^a^ (*n* = 2, 10)	ND^b^	0.64	0.63

*Note*: Sample sizes for the age subgroups are reported in the corresponding order in parentheses for each row. (lines 134–138): Inter‐rater variability was evaluated, for the APB muscle in 12 children (once in two children, twice in eight children and three times in two children) and for the ADM muscle in eight children (once in one child and twice in seven children). The age distributions were, for the APB muscle, two 5–9‐year‐olds and 10 10–17‐year‐olds; and for the ADM muscle two 5–9‐year‐olds and six 10–17‐year‐olds. ICC values > 0.75, interpreted as indicating good reproducibility, are shown in bold.

Abbreviations: ADM, abductor digiti minimi; APB, abductor pollicis brevis; CMAP, compound muscle action potential; MUNIX, motor unit number index; MUSIX, motor unit size index (μV).

^a^ Sum scores were obtained by adding the values obtained for the two muscles.

^b^ Not determined because measurements could not be performed by the inexperienced investigator.

## 4 Discussion

This study provides reference values for the MUNIX and associated variables in children of different age groups and estimates of the reproducibility of the measurements. Results highlight the age variability of theses indexes depending on the studied muscle and the importance of experience with this method to achieve reliable results.

In our participants, the APB MUNIX and APB CMAP increased significantly with age but no such increase was observed for the ADM muscle. The APB muscle is a fine motor muscle while the ADM is a hand strength muscle, with specific somatotopic spinal cord (gray matter) arrangements in both cases, so one would expect different motor neuron maturation trends in these two muscles. The only existing age‐specific MUNIX data for control children are those reported by Verma et al. [12]. In terms of the age groups used in our study, the median values reported by Verma et al. [12] for the eight control children included in their study are 154 (*n* = 2), 226 (*n* = 3), 219 (*n* = 2), and 334 (*n* = 1) for the APB MUNIX, versus 124, 170, 182, and 217 in our study, and 155, 236, 207, and 234 for the ADM MUNIX, versus 118, 181, 155, and 148 in our study. The comparisons for the MUSIX are similar, but the values reported by Verma et al. for the CMAP are consistently higher than ours (Table S2). With the exception of the CMAP however, Verma et al.'s values mostly fall within the corresponding ranges in our data, suggesting that there is a high level of physiological interindividual variability. Age‐specific trends should therefore be interpreted with caution and would require larger studies than ours to ascertain.

Large interindividual variability has also been evidenced by studies of MUNIX in adult controls. In repeat measurements performed on a group of 118 adults, Delmont et al. [19] obtained mean (standard deviation) APB MUNIX values of 168 (68) and then 163 (63) and mean ADM MUNIX values of 145 (41) and then 150 (43), with a similar level of variability for the CMAP and MUSIX (Table S2). In 38 adults under the age of 60 meanwhile, Neuwirth et al. [5] obtained a mean APB MUNIX of 177 (51) and a mean ADM MUNIX of 172 (49). These values are all within one standard deviation of the values measured in the different age groups in our study, suggesting once again that any association with age is weaker that the physiological variability of these measures between individuals of the same age. In contrast, the differences with patients with motor neuron diseases are much larger than the differences between age groups in control adults and children [3, 10, 12, 13].

In terms of reliability, our results are consistent with previous findings [19] that MUNIX measurements performed by an experienced investigator are highly reproducible. Also in agreement with Delmont et al.'s findings however, our data indicate that measurements performed by inexperienced investigators lack the necessary reproducibility, particularly in younger children. While the measurements performed among 10–17‐year‐olds were in all but one case found to be highly reproducible, those among 5–10‐year‐olds were all below the required threshold and measurements among under‐fives proved too difficult for the inexperienced investigator. The fact that the sum scores had higher ICCs may reflect regression to the mean. These results emphasize the relatively shallow learning curve associated with the MUNIX technique and the benefits of experience.

A major limitation of the study is that participants were recruited among patients who consulted for suspected neuromuscular disease and are perhaps not representative of the general pediatric population. The data were also cross‐sectional, rather than longitudinal from the same group of growing children. The other main limitations of the study are its small size relative to the variability of the studied measures, and the fact that repeat measurements could only be performed for a subset of the included children.

## 5 Conclusion

In this group of 60 control children between 3 and 17 years of age, the MUNIX technique performed by an experienced investigator was found to provide highly reproducible measures of motor units. The reference values provided for the different age groups should be helpful in everyday clinical practice. Larger studies are required to confirm the trends observed in the evolution of the different MUNIX parameters with age. The good reproducibility of MUNIX in experienced hands for children of all ages supports the use of these parameters as electrophysiological biomarkers for disease progression and potential treatment response.

## Author Contributions

**Christophe Boulay:** conceptualization, writing – review and editing, writing – original draft, formal analysis, investigation, methodology, software, validation, data curation, visualization. **Emilien Delmont:** writing – review and editing, data curation, software, validation, formal analysis, supervision. **Frédérique Audic:** writing – review and editing. **Cécile Halbert:** writing – review and editing. **Sébastien Pesenti:** writing – review and editing, formal analysis, writing – original draft. **Brigitte Chabrol:** writing – review and editing. **Béatrice Desnous:** writing – review and editing, formal analysis, writing – original draft. **Shahram Attarian:** writing – review and editing, supervision, project administration, conceptualization, methodology, validation, formal analysis, visualization.

## Ethics Statement

We confirm that we have read the journal's position on issues involved in ethical publication and affirm that this report is consistent with those guidelines.

## Conflicts of Interest

The authors declare no conflicts of interest.

## Supporting information


**Table S1.** Regression analysis.

**Table S2.** Comparison vs. literature data for reference values by age group in control children.

## Data Availability

The data that support the findings of this study are available on request from the corresponding author. The data are not publicly available due to privacy or ethical restrictions.
