# Supplementary material for: Motor Unit Number Index (MUNIX) in Control Children: Reference Values and Reliability
Source: Muscle Nerve. 2025 Jul 21;72(4):625–31. doi: 10.1002/mus.28470 (PMC12435161; doi:10.1002/mus.28470)
Supplement: Supplementary file 1 — Table S1. Regression analysis. [file MUS-72-625-s002.docx]

**Supporting Information**

**Table S1.** Regression analysis

|  | Univariable analysis | | | Multivariable analysis | | |
| --- | --- | --- | --- | --- | --- | --- |
|  | Adj. R^2^ | Slope | *p* | Adj. R^2^ | Slope | *p* |
| APB MUNIX |  |  |  |  |  |  |
| Age (years) | 0.20 | 6.5 [3.2–9.7] | < .001 | 0.19 | 1 [−9–11] | .83 |
| Height (cm) | 0.21 | 1.0 [0.5–1.5] | < .001 |  | 0.6 [−0.9–2.0] | .44 |
| Weight (kg) | 0.20 | 1.7 [0.4–4.0] | < .001 |  | 0.6 [−1.6–2.7] | .59 |
| Sex | −0.02 |  |  | – | – | – |
| APB MUSIX |  |  |  |  |  |  |
| Age (years) | 0.05 | – | – | – | – | – |
| Height (cm) | 0.03 | – | – |  |  |  |
| Weight (kg) | 0.03 | – | – |  |  |  |
| Sex | −0.02 | – | – |  |  |  |
| APB CMAP |  |  |  |  |  |  |
| Age (years) | 0.43 | 0.42 [0.30–0.52] | < .001 | 0.42 | 0.38 [−0.02–0.77] | .065 |
| Height (cm) | 0.35 | 0.06 [0.04–0.08] | < .001 |  | −0.02 [−0.07–0.04] | .52 |
| Weight (kg) | 0.40 | 0.11 [0.07–0.14] | < .001 |  | 0.05 [−0.04–0.13] | .28 |
| Sex | −0.02 |  |  | – | – | – |
| ADM MUNIX |  |  |  |  |  |  |
| Age (years) | −0.02 | – | – |  |  |  |
| Height (cm) | −0.02 | – | – |  |  |  |
| Weight (kg) | −0.02 | – | – |  |  |  |
| Sex | −0.03 | – | – |  |  |  |
| ADM MUSIX |  |  |  |  |  |  |
| Age (years) | 0.11 | – | – |  |  |  |
| Height (cm) | 0.09 | – | – |  |  |  |
| Weight (kg) | 0.09 | – | – |  |  |  |
| Sex | 0.002 | – | – |  |  |  |
| ADM CMAP |  |  |  |  |  |  |
| Age (years) | 0.11 | – |  |  |  |  |
| Height (cm) | 0.03 | – |  |  |  |  |
| Weight (kg) | 0.04 | – |  |  |  |  |
| Sex | 0.06 | – |  |  |  |  |

Regression coefficients (slopes) are presented as point estimate [95% confidence interval]

ADM, abductor digiti minimi; APB, abductor pollicis brevis; CMAP, compound muscle action potential (mV); MUNIX: motor unit number index; MUSIX: motor unit size index (µV)
