# Supplementary material for: Motor Unit Number Index (MUNIX) in Control Children: Reference Values and Reliability
Source: Muscle Nerve. 2025 Jul 21;72(4):625–31. doi: 10.1002/mus.28470 (PMC12435161; doi:10.1002/mus.28470)
Supplement: Supplementary file 2 — Table S2. Comparison vs. literature data for reference values by age group in control children. [file MUS-72-625-s001.docx]

**Supporting Information**

| **Table S2.** Comparison vs literature data for reference values by age group in control children | | | | | | | | |
| --- | --- | --- | --- | --- | --- | --- | --- | --- |
|  | < 5 year-olds | | 5–9 year-olds | | 10–15 year-olds | | 16–17 year-olds | |
|  | Median (IQR) | 5–95th %ile | Median (IQR) | 5–95th %ile | Median (IQR) | 5–95th %ile | Median (IQR) | 5–95th %ile |
| MUNIX |  |  |  |  |  |  |  |  |
| APB (*n* =16,15,11,8) | 124 (92–153) | 59–196 | 170 (127–201) | 107–224 | 182 (146–270) | 80–326 | 217 (192–243) | 128–272 |
| Verma (*n* = 2, 3, 2, 1) | 154 |  | 226 |  | 219 |  | 314 |  |
| Delmont (118 adults) | 168 (68), 163 (63) | | | | | | | |
| Neuwirth (38 adults) | 177 (51) | | | | | | | |
| ADM (*n* = 5,12,16,8) | 118 (117–139) | 99–217 | 181 (147–229) | 91–280 | 155 (135–193) | 93–250 | 148 (137–164) | 108–181 |
| Verma (*n* = 2, 3, 2, 1) | 155 |  | 236 |  | 207 |  | 234 |  |
| Delmont (118 adults) | 145 (41), 150 (43) | | | | | | | |
| Neuwirth (38 adults) | 172 (49) | | | | | | | |
| MUSIX |  |  |  |  |  |  |  |  |
| APB (*n* = 16,15,11,8) | 45 (40–48) | 29–71 | 52 (39–59) | 31–70 | 52 (40–68) | 32–86 | 52 (49–61) | 42–74 |
| Verma (*n* = 2, 3, 2, 1) | 62 |  | 47 |  | 67 |  | 55 |  |
| Delmont (118 adults) | 61 (16), 63 (17) | | | | | | | |
| ADM (*n* = 5,12,16,8) | 45 (41–55) | 34–56 | 59 (44–62) | 32–69 | 63 (53–75) | 35–85 | 63 (53–68) | 43–89 |
| Verma (n = 2, 3, 2, 1) | 73 |  | 49 |  | 67 |  | 58 |  |
| Delmont (118 adults) | 71 (15), 69 (14)s | | | | | | | |
| CMAP |  |  |  |  |  |  |  |  |
| APB (*n* = 16,15,11,8) | 5.7 (4.1–7.5) | 2.8–7.9 | 8.1 (6.7–9.0) | 4.6–11.1 | 10.1 (7.0–11.4) | 6.2–14.4 | 11.6 (10.2–12.6) | 8.2–13.8 |
| Verma (*n* = 2, 3, 2, 1) | 9.6 |  | 11.6 |  | 14.6 |  | 17.2 |  |
| Delmont (118 adults) | 9.6 (2.9), 9.7 (2.6) | | | | | | | |
| Neuwirth (38 adults) | 10.1 (2.5) | | | | | | | |
| ADM (*n* = 5,12,16,8) | 6.6 (5.3–7.5) | 4.3–7.6 | 9.0 (7.9–10.7) | 6.1–12.6 | 9.4 (8.0–11.0) | 6.5–12.3 | 9.3 (7.6–9.7) | 6.9–11.1 |
| Verma (*n* = 5,12,16,8) | 11.4 |  | 13.4 |  | 13.6 |  | 13.5 |  |
| Delmont (118 adults) | 9.9 (2.2), 10.1 (2.2) | | | | | | | |
| Neuwirth (38 adults) | 10.8 (2.0) | | | | | | | |
| Patient numbers for the age subgroups are reported in the corresponding order in parentheses for each row | | | | | | | | |
| ADM, abductor digiti minimi; APB, abductor pollicis brevis; CMAP, compound muscle action potential (mV); MUNIX: motor unit number index; MUSIX: motor unit size index (µV) | | | | | | | | |
